# CNTs/Fe-BTC Composite Materials for the CO_2_-Photocatalytic Reduction to Clean Fuels: Batch and Continuous System

**DOI:** 10.3390/molecules28124738

**Published:** 2023-06-13

**Authors:** Elizabeth Rojas García, Gloria Pérez-Soreque, Ricardo López Medina, Fernando Rubio-Marcos, Ana M. Maubert-Franco

**Affiliations:** 1 Área de Ingeniería Química, Departamento de Ingeniería de Procesos e Hidráulica, Universidad Autónoma Metropolitana-Iztapalapa, Mexico City 09340, Mexico; 2 Laboratorio de Catálisis y Materiales, ESIQIE-Instituto Politécnico Nacional Zacatenco, Mexico City 07738, Mexico; 3 Área de Química de Materiales, Departamento de Ciencias Básicas e Ingeniería, Universidad Autónoma Metropolitana-Azcapotzalco, Mexico City 02200, Mexico; 4 Área de Procesos de la Industria Química, Departamento de Energía, Universidad Autónoma Metropolitana-Unidad Azcapotzalco, Mexico City 02200, Mexico; 5 Departamento de Electrocerámica, Instituto de Cerámica y Vidrio, CSIC, Kelsen 5, 28049 Madrid, Spain; 6 Escuela Politécnica Superior, Universidad Antonio de Nebrija, C/Pirineos 55, 28040 Madrid, Spain

**Keywords:** Fe-BTC, CNTs/Fe-BTC, CO_2_-photocatalytic reduction, CO_2_, clean fuels, MWCNTs, SWCNTs

## Abstract

CNTs/Fe-BTC composite materials were synthesized with the one-step solvothermal method. MWCNTs and SWCNTs were incorporated in situ during synthesis. The composite materials were characterized by different analytical techniques and used in the CO_2_-photocatalytic reduction to value-added products and clean fuels. In the incorporation of CNTs into Fe-BTC, better physical–chemical and optical properties were observed compared to Fe-BTC pristine. SEM images showed that CNTs were incorporated into the porous structure of Fe-BTC, indicating the synergy between them. Fe-BTC pristine showed to be selective to ethanol and methanol; although, it was more selective to ethanol. However, the incorporation of small amounts of CNTs into Fe-BTC not only showed higher production rates but changes in the selectivity compared with the Fe-BTC pristine were also observed. It is important to mention that the incorporation of CNTs into MOF Fe-BTC allowed for increasing the mobility of electrons, decreasing the recombination of charge carriers (electron/hole), and increasing the photocatalytic activity. In both reaction systems (batch and continuous), composite materials showed to be selective towards methanol and ethanol; however, in the continuous system, lower production rates were observed due to the decrease in the residence time compared to the batch system. Therefore, these composite materials are very promising systems to convert CO_2_ to clean fuels that could replace fossil fuels soon.

## 1. Introduction

Approximately 85% of the world’s primary energy is supplied from fossil fuels (oil, coal, etc); however, it is expected that in the near future, the use of these will be limited by the emission controls that have been agreed upon by the United National Organization (ONU). The combustion of fossil fuels (oils, carbon, etc.) emits large amounts of greenhouse gases such as CO_2_, NO_x_, and SO_x_, among others [1,2]. However, carbon dioxide (CO_2_) is the one that is emitted to the greatest extent into the atmosphere, and it is the main one responsible for the global warming of the planet that is generating serious consequences such as severe weather changes, melting of the poles, desertification of forests, etc. [3].

Currently, several processes are being investigated to reduce the concentration of CO_2_ in the atmosphere such as absorption in amines [4,5], capture using solid materials as adsorbents [6,7,8,9], and, very recently, its conversion to value-added products (formic acid, formaldehyde, and acetaldehyde, among others) and clean fuels (hydrogen, methane, methanol, ethanol, etc.) through the photocatalytic reduction reaction of CO_2_ [10,11,12,13,14]. The CO_2_-photocatalytic reduction reaction is a process that allows CO_2_ to be converted to value-added products and clean fuels through the use of a photocatalyst and light in the visible or ultraviolet region [15,16]. This process is extremely interesting since it simulates the natural photosynthesis of plants [17]. However, the conversion of carbon dioxide is a very difficult process since it is a linear molecule and very stable, with a C=O binding energy of 750 kJ/mol, which is higher than that of C-C (336 kJ/mol), C-O (327 kJ/mol), and C-H (411 kJ/mol), which implies that large amounts of energy are necessary for it to react with other compounds. In addition, to reduce the CO_2_ molecule to CO_2_^−^, a reduction potential of −1.9 eV is necessary [11,18].

Actually, several catalysts (ZnMn_2_O_4_, ZIFs, Co_3_O_4_, and Zn_2_GeO_4_, among others) are being studied in the CO_2_-photocatalytic reduction reaction [19,20,21]; however, the production rate towards value-added products and clean fuels is still very slow. For this reason, it is necessary to search for other materials that allow obtaining a better production rate, such as metal–organic frameworks (MOFs). MOFs are hybrid organic–inorganic structures made up of metal ions or clusters and multidentate organic bonds connected to metal nodes in one, two, or three dimensions, in which divalent or trivalent carboxylic acids are usually used to form structures with zinc, chromium, copper, aluminum, and zirconium, among others [22]. These materials have been rapidly developed in recent years due to the variety of their structures, as well as their easy adaptation and different applications such as catalysis, separation, gas storage, carbon dioxide capture, controlled drug release, luminescence, dye photocatalytic degradation, CO_2_-photocatalytic reduction, etc. [23,24,25,26,27,28].

MOFs have not only been shown to have a high surface area and controllable porosity but also have demonstrated excellent photocatalytic properties [29]. The latest studies have been focused on modifying the synthesis method mainly by green methods (hydrothermal, microwave, mechanochemical, etc.), or change in the band gap through the incorporation of metals, metal oxides, combination of organic ligands and metallic clusters, and carbonaceous materials such as carbon nanotubes (CNTs) [30,31,32,33].

The MOF Fe-benzene-1,3,5-tricarboxylate (Fe-BTC) is made up of Fe^3+^ ions and BTC linkers. Its composition is expressed by the C_9_H_3_FeO_6_ empirical formula and its mass content of iron and carbon is 21% and 41%, respectively. The structure of Fe-BTC is not well known due to its poor crystallinity, which is described in the literature as a disordered material or semi-amorphous [34,35]. This MOF has been studied in the separation of organic compounds in the liquid phase, oxidation reactions, dye adsorption, gases separation/adsorption, sensing, As(III) oxidation/adsorption process, and dye photocatalytic degradation, among other applications [36,37,38,39,40]. Our group has studied this material as an adsorbent in heavy metals, dye and drug adsorption, etc. [29,41,42].

CNTs can be used as photosensitizers or band gap modifiers. Furthermore, due to their excellent optical and electron transfer properties, carbonaceous materials have been extensively studied to improve the photoactivity of some semiconductors [43,44,45]. Several investigations have demonstrated that the incorporation of CNTs into MOFs can improve the physicochemical and optical properties of MOF pristine. Lin et al. [46] showed that the incorporation of CNTs into MOF UiO-66 (Zr) not only significantly improved the adsorption capacity for acid dyes but also enabled these UiO-66 derivatives to exhibit photocatalytic activity under visible light irradiation. Other recent research demonstrated that the addition of CNTs to MOF-808 significantly improved the photocatalytic activity in the treatment of pharmaceutical and agrochemical wastewaters [47].

In the present work, CNTs/Fe-BTC composite materials were synthetized with the one-step solvothermal method, where CNTs were added during the synthesis process. The composite materials synthetized were characterized by different analytical techniques and evaluated in the CO_2_-photocatalytic reduction reaction using two reaction systems (batch and continuous) under visible or UV irradiation and mild reaction conditions (room temperature and pressure).

## 2. Results and Discussion

**Figure 1:**
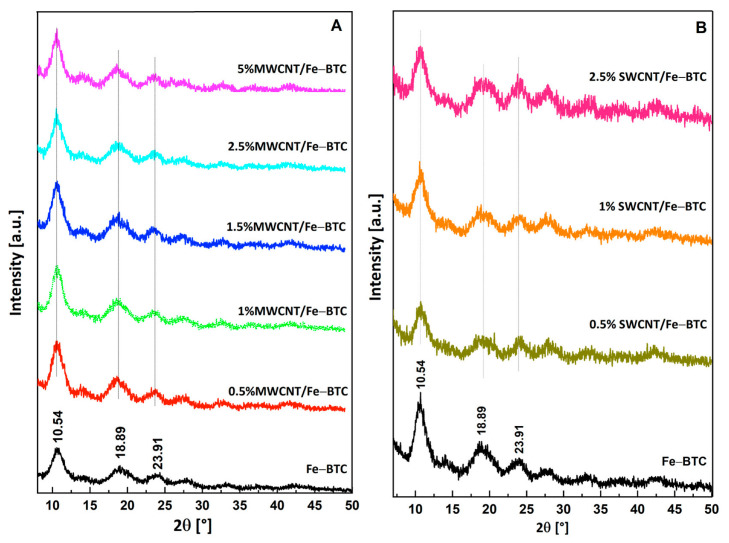
(**A**) XRD patterns of the MOF Fe-BTC and of the composite materials with different percentages of MWCNT, and (**B**) Spectra Raman of MWCNT, MOF Fe-BTC, and composite materials.

Figure 1 shows the power X-ray diffraction patterns (PXRD) of MOF Fe-BTC pristine and composite materials. In the PXRD of Fe-BTC pristine, broad peaks were observed at 2θ = 10.54, 18.89, and 23.91º—characteristics of MOF Fe-BTC, according to the literature [26,27]. These same peaks were observed in both series of the composite materials, indicating that the incorporation of CNTs does not modify the structure of the MOF. Due to the small amount of CNTs added to the composite materials, peaks were not shown in the diffraction patterns observed.

**Figure 2:**
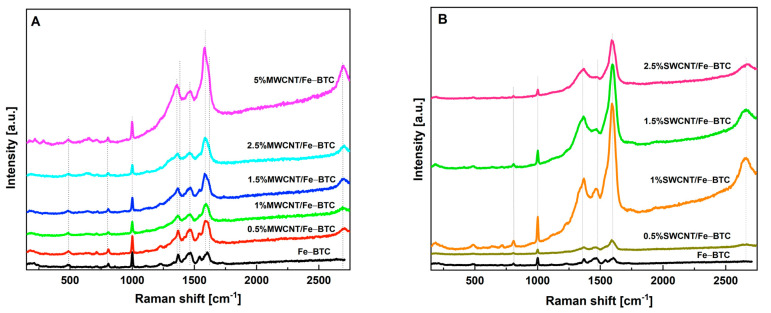
Raman spectra of all the materials, (**A**) MWCNTs’ series, and (**B**) SWCNTs’ series.

The Raman analysis was used for determining the presence of the CNTs in the composite materials. In the Raman spectra of the composite materials, bands corresponding to CNTs and the organic ligand of MOF were mainly observed. Raman spectra of the MOF Fe-BTC pristine and composite materials are shown in Figure 2. In the case of the Fe-BTC spectrum, it can be divided into two zones: the first in the range of 1750–700 cm^−1^, which shows bands characteristic of the vibrations corresponding to the organic ligand, and the second zone is in the range of 700–400 cm^−1^, corresponding to the vibrations of the secondary basic units or SBUs (metal cluster) and the interaction between the SBUs and organic ligands [26,42]. Additionally, other bands at 1377, 1608, and 2693 cm^−1^ were observed in the composite materials, corresponding to carbon nanotubes. The bands at 1377 and 1608 cm^−1^ are characteristics of the D band (disorder) and G band (graphite), respectively. The band at 2693 cm^−1^ corresponds to the 2D band (second-order harmonic). Thus, the presence of these three bands indicates the presence of CNTs in the composite materials.

**Figure 3:**
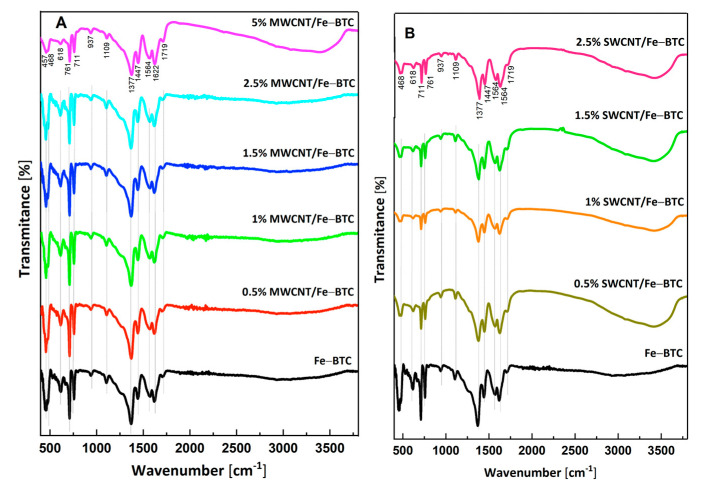
FTIR spectra of the materials, (**A**) MWCNTs’ series, and (**B**) SWCNTs’ series.

Figure 3 shows the FTIR spectra of the Fe-BTC and composite materials. All materials’ FTIR spectra showed a broad band between 2700 and 3600 cm^−1^, corresponding to the symmetric and asymmetric vibrations of the O-H bond from the water molecules absorbed in the pores of the MOF [48]. Additionally, FTIR spectra are observed as the characteristic bands of the organic ligand and metallic clusters. For example, bands at 1447, 1564 y 1622 cm^−1^ are characteristics of the C=C vibrational group of the benzenic ring of trimesic acid. Additionally, other vibrational bands are observed between 1300 and 400 cm^−1^, which are attributed to symmetric stretching vibration of the carboxylate group (C-O). Finally, it is possible observed bands at 488 and 616 cm^−1^ are characteristics of the interaction between the organic ligand and metallic cluster (O_2_-Fe) [49].

**Figure 4:**
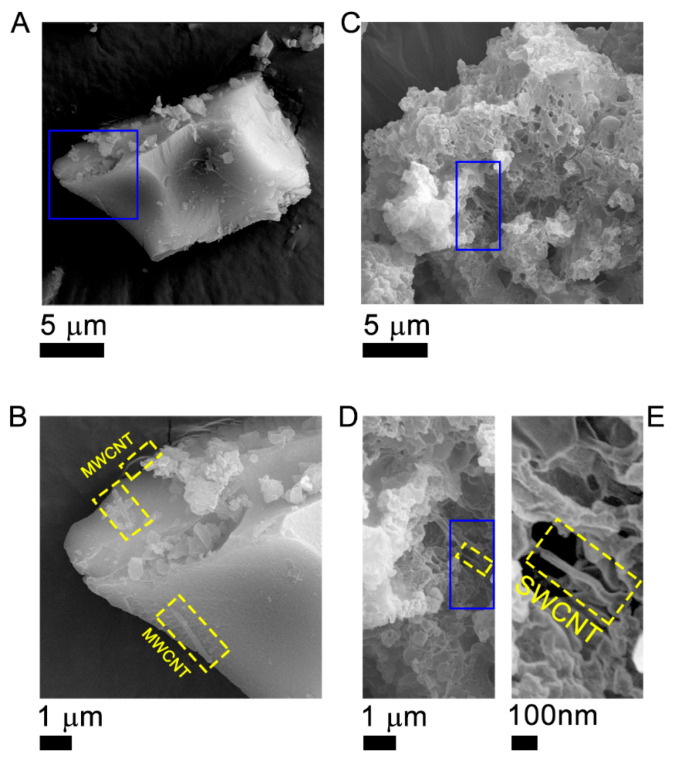
Identification of the morphology of the CNT/Fe-BTC composite materials. Morphology of (**A**,**B**) 1.5% MWCNT/Fe-BTC and (**C**–**E**) 1.5% SWCNT/Fe-BTC composite materials.

**Figure 5:**
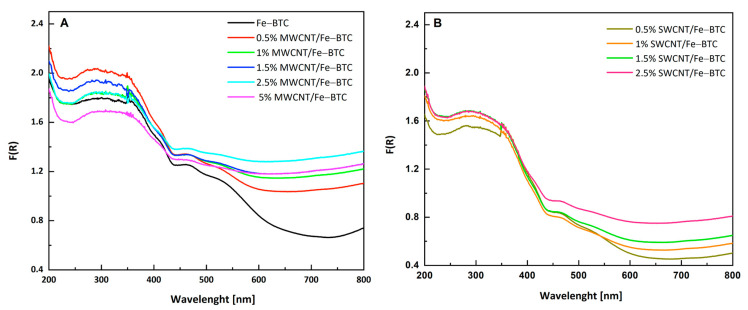
UV-Vis absorption spectra of (**A**) MWCNT series and (**B**) SWCNT series.

To understand the distribution of each component and clarify the dispersion state of the carbon nanotubes in the composite materials, FE-SEM characterization has been carried out on the 1.5% MWCNT/Fe-BTC and 1.5% SWCNT/Fe-BTC samples, as can be observed in Figure 4. In both composite systems, the typical morphology of Fe-BTC can be observed (Figure S1), which consists of irregular particles with sizes in the range of micrometers (Figure 4A,C) [35]. More interestingly, at higher magnifications (Figure 4D–E and Figure 5B), it is observed that the CNTs have been adequately incorporated into the matrix of the MOF (Figure S2). This fact allows us to infer that the incorporation process of both MWCNTs and SWCNTs generates the appearance of a synergy between CNTs and the MOF matrix.

Figure 5 shows UV-Vis absorption spectra of both series. All the materials showed a broad absorption band from 250 to 350 nm, characteristic of π–π* transitions of the organic ligands [50]; another absorption band for all samples in the visible region (380–700 nm) was observed, which can be ascribed to the spin-allowed d–d electronic transitions of the trivalent metal ions (Fe^3+^). The band gap of the materials was obtained using the Tauc equation (Table 1 and Figure S3). As can be seen in Table 1, all materials are capable of absorbing photons with energies in the visible region. The incorporation of MWCNT in MOF Fe-BTC shows a decrease in the band gap due to the intimate interaction between them (Table 1). Whereas, for the SWCNT series, very small changes in the band gap were observed by increasing the percentage of carbon nanotubes. Other authors observed this same behavior; for example, Samy et al. [47] showed that the addition of CNTs to MOF-808 reduced the band gap, resulting in higher photocatalytic performance in the treatment of pharmaceutical and agrochemical wastewaters.

**Figure 6:**
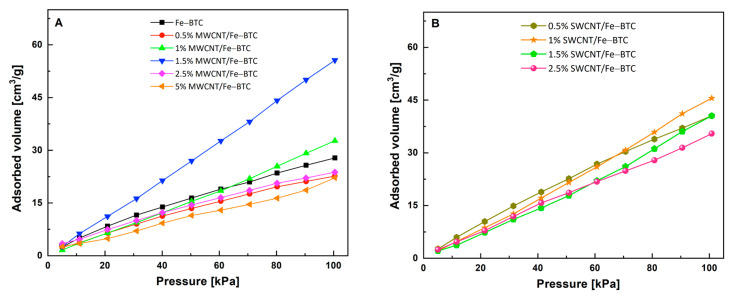
CO_2_ adsorption isotherm at 298 K and low pressure for Fe-BTC pristine and composite materials, (**A**) MWCNT series, and (**B**) SWCNT series.

Narayanan et al. [15] mentioned that the interaction of the CO_2_ molecule with the semiconductor surface through oxygen or carbon atoms modifies the degree of reactivity in the molecule through surface reactions, this being a key step in some chemical reactions. For example, a fundamental step in the CO_2_-photocatalytic reduction reaction is the activation of CO_2_. CO_2_ adsorption in the MOF surface and the electrons’ surface generated when they are caught up by the CO_2_ molecule allows for reducing CO_2_ to CO_2_^−^ radical. The formation of CO_2_^−^ radical allows for modifying its high stability by changing its linear geometry to bent. Figure 6 shows the CO_2_ adsorption isotherms at 298 K and atmospheric pressure for Fe-BTC pristine and composite materials. Table 1 shows the CO_2_-adsorption maximum capacity at 298 K and 1 atm. This analysis was carried out to quantify the amount of CO_2_ that the materials can adsorb on their surface, which will give us important information about their behavior in the photocatalytic reaction. As can be observed in Table 1, the materials that show the highest adsorption capacities are 1.5% MWCNT/Fe-BTC at 55.6 cm^3^/g, followed by 1% MWCNT/Fe-BTC at 32.7 cm^3^/g. Meanwhile, SWCNT series–composite materials display the highest CO_2_-adsorption capacity, if we compare with MWCNT series—the 1% SWCNT/Fe-BTC sample being with the highest CO_2_-adsorption capacity of this series. Activation is a crucial step in the CO_2_-photocatalytic reduction reaction, given that when this is adsorbed on the surface of the composite material, it takes blending-type geometries that significantly decrease its reduction potential [16].

Table 2 shows the production rate and product selectivity formed in the CO_2_-photocatalytic reduction reaction for both systems (batch and continuous) using visible or UV irradiation. As can be observed, in the batch system and visible irradiation, Fe-BTC is selective to ethanol, while for the incorporation of MWCNT in MOFs, changes in the selectivity to methanol were observed, except for the sample 1% MWCNT/Fe-BTC being selective to ethanol. The sample with the highest production towards methanol is 0.5% MWCNT/Fe-BTC with 1566 µmol/g*h, followed by 1.5% MWCNT/Fe-BTC with 1440 µmol/g*h, both are superior to Fe-BTC, while the 1% MWCNT/Fe-BTC composite material presents a higher production towards ethanol than the one obtained for Fe-BTC. This same behavior was observed in the composite materials based in SWCNT. The sample of 1.5% SWCNT/Fe-BTC shows the highest selectivity to methanol, while in the 1% MWCNT/Fe-BTC, the highest selectivity to ethanol was observed. Several of the composite materials (1% MWCNT/Fe-BTC, 1.5% MWCNT/Fe-BTC, 1% SWCNT/Fe-BTC, and 1.5% SWCNT/Fe-BTC) and Fe-BTC pristine were tested using the batch system and UV irradiation as shown in Table 2. As can be observed, a change in the selectivity of the products in the Fe-BTC pristine was observed, this being more selective to methanol. Meanwhile, all composite materials showed to be selective to methanol. Comparing the results obtained in the continuous system with the batch system using visible light as a source irradiation, a decrease in the production rate was observed (for example, 1.5% MWCNT/Fe-BTC or 1.5% SWCNT/Fe-BTC). This could be due to the decrease in the residence time in the continuous system. Additionally, Fe-BTC pristine and composite materials showed to be selective to methanol and the presence of formic acid is more important. Some MOFs are known by their low stability in an aqueous medium; thus, in Figure S4, the Raman spectra of the Fe-BTC pristine and composite materials used in the reaction are shown. All composite materials showed bands in the range of 700–400 cm^−1^ and 1750–700 cm^−1^—characteristic of the vibrational groups corresponding to the interaction between the SBU and organic ligand and the presence of bands corresponding to the organic ligand, respectively. The presence of these bands is indicative of a high stability of the composite materials in an aqueous medium.

CO_2_-photocatalytic reactions are complex multistep reactions where the selectivity to desired products can be affected by many *thermodynamic* and *kinetic* factors. Fu et al. [18] mentioned that any *thermodynamic* factors are the photon energy and CB position (i.e., activation of the semiconductor and formation of photogenerated electrons and holes, this being the critical initial step in the CO_2_-photocatalytic reaction), and any *kinetic* factors are light intensity, surface catalytic active sites, separation of photogenerated charge carriers, and adsorption/desorption of reactants/intermediates. Photon energy and light intensity are fundamental factors that determine whether a semiconductor can absorb photons or the number of photogenerated electrons and holes, respectively. Both types of factors affect the product selectivity and reaction rate. Thus, in our case, changes in the band gap of MOF pristine, photon energy (visible and UV light) that generates the charge carriers, the reaction system (batch or continuous), properties of the catalysts, CO_2_ adsorption, and other factors affect the production rate and product selectivity, as can be observed in Table 2.

**Figure 7:**
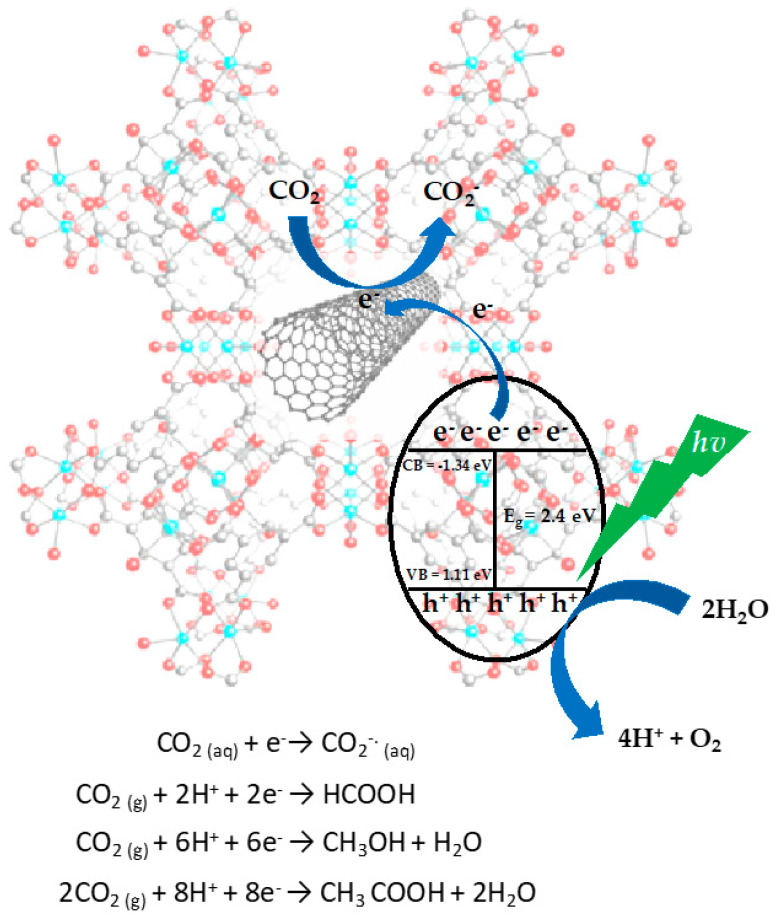
Possible reaction mechanism for the photocatalytic reduction reaction of CO_2_ using the composite materials.

**Figure 8:**
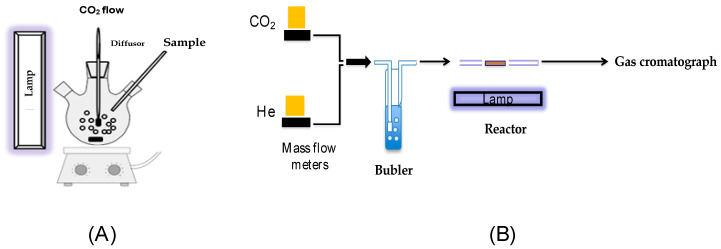
Reaction system for the photocatalytic reduction of CO_2_, (**A**) batch system, and (**B**) continuous system.

Figure 7 shows a possible charge transfer mechanism using the composite materials (CNT/Fe-BTC). Regarding our results obtained from the UV-Vis analysis, Fe-BTC and composite materials showed that these materials can be activated in the visible region. Once the charge carriers (electron–hole) are formed, the conduction band electrons are trapped by the carbon nanotubes due to their high capacity to disperse electrons. The latter makes it possible to reduce the recombination of charge carriers photogenerated. Once the CO_2_ molecule is adsorbed in the surface of the material, it is reduced to later carry out the chemical reaction. Meanwhile, the holes carry out the oxidation of water to H^+^ and O_2_. The protons, electrons, and adsorbed CO_2_ react to form formic acid, ethanol, and methanol, as can be observed in Figure 8 [15].

## 3. Materials and Methods

### 3.1. Materials

All materials were used without any treatment. Iron nitrate nonahydrate (Fe(NO_3_)_3_ 9H_2_O, Sigma-Aldrich, Rahway, NJ, USA, >98%), N,N-dimethylformamide (DMF, Merck, Rahway, NJ, USA, 99.5%), trimesic acid (C_9_H_6_O_6_, Sigma-Aldrich, 95%), multi-wall carbon nanotubes (MWCNT, D × L 110–120 nm × 5–9 µm, Sigma Aldrich, >90%), single-wall carbon nanotubes (SWCNT, 0.7–1.1 nm, Sigma Aldrich, >77%), ethanol (Sigma-Aldrich, 99.5%), and anhydrous methanol (Sigma-Aldrich, 99.8%) were used.

### 3.2. Functionalization of the CNTs

The CNTs, before being incorporated into the MOFs, were functionalized following the following methodology: 500 mg of CNTs was added to 50 mL of a mixture of H_2_SO_4_/HNO_3_ in a 1:1 molar ratio. This mixture was kept in a sand bath with vigorous agitation at 80 °C for 6 h. After this time, the mixture was cooled to room temperature and diluted in 1 L of deionized water, then filtered and washed with deionized water up to a pH of 7.0. Finally, the solid obtained was dried in an oven at 100 °C for 24 h.

### 3.3. Synthesis of Composite Materials

The composite materials were synthesized with the one-step solvothermal method, where the functionalized CNTs were incorporated during the synthesis process [35]. Briefly, 3.52 g of iron nitrate nonahydrate was added to 30 mL of DMF. Then, 1.76 g of trimesic acid and the amount of CNTs corresponding to the percentage to be synthesized (0.5, 1, 1.5, 2.5, and 5 wt.%) were added. This dissolution was placed in an ultrasonic bath for 5 min; after this time, 30 mL of ethanol and 30 mL of deionized water were added, and it was kept for 35 min more in an ultrasonic bath. The previous solution was placed in a sand bath with continuous agitation at 85 °C for 24 h. The solid obtained was filtered and washed with acetone for 24 h (3X). Finally, the solid obtained was dried at 80 °C for 24 h. This same methodology was used to obtain the Fe-BTC pristine.

### 3.4. Characterization of Materials

The materials synthetized were characterized by different analytical techniques such as X-ray diffraction (X’Pert PRO, Philips, Almelo, The Netherlands) with a CuKα anode (λ = 1.54178 Å) and a 0.02°/min step size; Raman spectroscopy (In Via, Renishaw, Gloucestershire, United Kingdom) using a green laser (532 nm) and five accumulatios of 10 s at room temperature; and N_2_-adsorption/desorption isotherms at 77 K were obtained with Belsorp equipment (BelJapan, Osaka, Japan). Prior to the analysis, samples were degassed at 80 ºC for 16 h in a nitrogen flow. BET surface areas were obtained from the adsorption isotherms (0.05 < P/P_0_ < 0.27), taking a value of 0.164 nm^2^ for the cross-section of the adsorbed N_2_ molecule. Field Emission Electron Microscopy (S-4700 FESEM, Hitachi, Tokyo, Japan) had a resolution from 1.5 nm to 15 kV and an acceleration voltage of 20 kV; UV-Vis spectroscopy (Cary 100, Agilent Technologies, Santa Clara, CA, USA) involved a wavelength range from 190 to 800 nm. The band gap was determined using the Tauc equation, [(F(R)*h**ν**)^1/2^], where F(R) is the Kubelka Munk function, h is the Planck constant, and **ν** is the light frequency.

### 3.5. Photocatalytic Evaluation

For the photocatalytic evaluation, two reaction systems (batch and continuous) were used. Batch system (Figure 8A): In total, 25 mg of the catalyst was added into a three-neck quartz reactor with 40 mL of deionized water. After, this dissolution was bubbled with CO_2_ (99.8% purity) through a diffuser at a constant flow of 2.8 mL/min for 30 min, without light irradiation. Subsequently, the reactor was irradiated with visible or UV light. After, samples were taken at different times (0, 10 min, 30 min, 1, 2, 3, 4, 5, 6, 7, and 8 h), taking aliquots of 0.5 mL and passing the sample through a filter of 0.45 μm (PTFE acrodisk). Finally, the reaction products were analyzed in a gas chromatograph (Agilent Technologies, HP6890) equipped with a flame ionization detector (FID) and a DB-Wax capillary column (Crosslinked Methyl Siloxane, 30 m × 0.25 mm × 0.25 μm thickness). Previously, to determine the concentration of the products in the samples, the gas chromatograph was calibrated with the reference reagents, such as methanol, ethanol, formic acid, and formaldehyde. Continuous system (Figure 8B): Prior to the analysis, the samples were pre-treated with a nitrogen flow at 80 °C for 16 h, in order to eliminate residual solvents from the synthesis. The system is provided with a glass saturator where a flow of 10 mL/min of CO_2_ was bubbled into deionized water. The water-saturated CO_2_ was passed through a quartz fixed-bed tubular reactor, which contained 25 mg of the catalyst and (visible or UV) light irradiation was turned on. At the end of the reactor’s activity, a cooler is placed in order to condense the products of the reaction. Samples were taken every hour during 8 h of the reaction. Finally, samples were analyzed in the same gas chromatograph used in the batch system.

## 4. Conclusions

In conclusion, we could observe that the incorporation of CNTs in MOF Fe-BTC does not modify the structure of the Fe-BTC pristine. In addition, in the SEM images, it was observed that the CNTs embedded into Fe-BTC particles, probably indicating a close interaction (synergy) between them. This close interaction allowed for obtaining more active and selective composite materials towards the formation of ethanol and methanol due to an increase in the mobility of electrons and therefore a decrease in the recombination of the photogenerated charge carriers. Additionally, in the batch system, a high production rate was obtained compared with the continuous system; the reactants have less time to react in the latter. Additionally, composite materials showed to be stable in an aqueous medium. Therefore, these materials showed to be very promising systems in the photocatalytic conversion of CO_2_ to clean fuels such as methanol and ethanol.

## Figures and Tables

**Table 1:** CO_2_^−^ adsorption capacity and band gap of both series of materials.

Samples	Band Gap (eV)	CO_2_ Adsorption Capacity (cm^3^/g)
Fe-BTC	2.49	27.8
0.5% MWCNT/Fe-BTC	2.41	22.6
1% MWCNT/Fe-BTC	2.61	32.7
1.5% MWCNT/Fe-BTC	2.31	55.6
2.5% MWCNT/Fe-BTC	2.13	23.7
5% MWCNT/Fe-BTC	2.10	22.1
0.5% SWCNT/Fe-BTC	2.41	40.5
1% SWCNT/Fe-BTC	2.52	45.5
1.5% SWCNT/Fe-BTC	2.50	40.5
2.5% SWCNT/Fe-BTC	2.41	35.4

**Table 2:** Production rate and selectivity of products formed in the photoreduction of CO_2_ for all materials.

Sample	Production Rate (μmol/g*h)			Selectivity (%)		
	Methanol	Ethanol	Formic Acid	Methanol	Ethanol	Formic Acid
**Batch system—visible light**						
Fe-BTC	88.5	292.9	0	23.2	76.8	0.0
0.5% MWCNT/Fe-BTC	1566	671.2	25	69.2	29.7	1.1
1% MWCNT/Fe-BTC	273	1569.3	0	14.8	85.2	0.0
1.5% MWCNT/Fe-BTC	1443.3	650.8	600.5	53.6	24.2	22.3
2.5% MWCNT/Fe-BTC	459.4	205.8	15	67.5	30.3	2.2
5% MWCNT/Fe-BTC	883.6	282.2	0	75.8	24.2	0.0
0.5% SWCNT/Fe-BTC	530.8	241	0	68.8	31.2	0.0
1% SWCNT/Fe-BTC	192.8	602	0	24.3	75.7	0.0
1.5% SWCNT/Fe-BTC	2631.8	700.8	0	79.0	21.0	0.0
2.5% SWCNT/Fe-BTC	188.4	95.3	0	66.4	33.6	0.0
**Batch system—UV light**						
Fe-BTC	178.3	144	0	55.3	44.7	0.0
1% MWCNT/Fe-BTC	3163	1104	0	74.1	25.9	0.0
1.5% MWCNT/Fe-BTC	163.9	132	0	55.4	44.6	0.0
1% SWCNT/Fe-BTC	425.6	126.8	136.6	61.8	18.4	19.8
1.5% SWCNT/Fe-BTC	194.7	144	0	57.5	42.5	0.0
**Continuous system—visible light**						
Fe-BTC	476.9	159.1	137.4	61.7	20.6	17.8
1% MWCNT/Fe-BTC	195.6	140.9	186.7	37.4	26.9	35.7
1.5% MWCNT/Fe-BTC	163.9	96.4	0	63.0	37.0	0.0
1% SWCNT/Fe-BTC	983	180.4	121.5	76.5	14.0	9.5
1.5% SWCNT/Fe-BTC	451	205.2	76.8	61.5	28.0	10.5

## Data Availability

Not applicable.

## Supplementary Materials

The following supporting information can be downloaded at: https://www.mdpi.com/article/10.3390/molecules28124738/s1, The following supporting information can be downloaded at: https://www.mdpi.com/article/10.3390/molecules28124738/s1, Figure S1: Morphological characterization of the Fe-BTC materials used as matrix in the CNT/Fe-BTC composite materials; Figure S2: Morphological characterization of the single-walled carbon nanotubes (SWCNTs) and multi- walled carbon nanotubes (MWCNTs) used as filler in the CNT/Fe-BTC composite materials; Figure S3: Determination of Band gap through of the Tauc equation; Figure S4: Raman Spectra of the MOF Fe-BTC and composite materials used in the CO_2_- photocatalytic reduction reaction for both series, (A) MWCNT serie, and (B) SWCNT serie.

## Sample Availability

Samples of the compounds are not available from the authors upon request.
