# Supplementary material for: CNTs/Fe-BTC Composite Materials for the CO2-Photocatalytic Reduction to Clean Fuels: Batch and Continuous System
Source: Molecules. 2023 Jun 13;28(12):4738. doi: 10.3390/molecules28124738 (PMC10304569; doi:10.3390/molecules28124738)
Supplement: Supplementary file 1 [file molecules-28-04738-s001.zip › molecules-2327906-supplementary.pdf]

Supporting Information

CNTs/Fe-BTC Composite Materials for the CO2-Photocatalytic Reduction to Clean Fuels: Batch and Continuous System

Elizabeth Rojas García[1], Gloria Pérez-Soreque[2], Ricardo López Medina[3], Fernando Rubio-Marcos [4,5], Ana M. Maubert-Franco[2*]*

[1] *Área de Ingeniería Química, Departamento de Ingeniería de Procesos e Hidráulica, Universidad Autónoma Metropolitana-Iztapalapa, Mexico City 09340. México.*

[2] *Área de Química de Materiales, Departamento de Ciencias Básicas e Ingeniería, Universidad Autónoma Metropolitana-Azcapotzalco, Mexico City 02200, Mexico*

[3] *Área de Procesos de la Industria Química, Departamento de Energía, Universidad Autónoma Metropolitana-Unidad Azcapotzalco, Mexico City 02200, Mexico*

[4] *Departamento de Electrocerámica, Instituto de Cerámica y Vidrio, CSIC, Kelsen 5, 28049 Madrid, España*

[5] *Escuela Politécnica Superior, Universidad Antonio de Nebrija, C/Pirineos, 55, 28040 Madrid, España*

Corresponding Authors

*E-mail: erg@xanum.uam.mx (E.R.-G);

*E-mail: amf@azc.uam.mx (A.M.M.-F.)

S1. Basic identification of the morphology of Fe-BTC matrix material.

To validate the efficiency of the synthesis strategies on the MOF-matrix material, the microstructure of Fe-BTC sample is characterized by using a Field Emission Scanning Electron Microscope, FE-SEM (Hitachi S-4700). It is found that the morphology of Fe-BTC consists of irregular particles with sizes in the range of micrometers (see Figure S1A). An observation of Fe-BTC at higher magnifications (**Fig. S1B**)) shows particles with irregular surfaces like sheets characteristic of the fractured FE-BTC. Finally, to identify the Min composition of Fe species on FE-BTC, an EDS mapping was carried out on Figure 1SA. As expected, Figures 1SC show the main element of FE-BTC and their distribution.

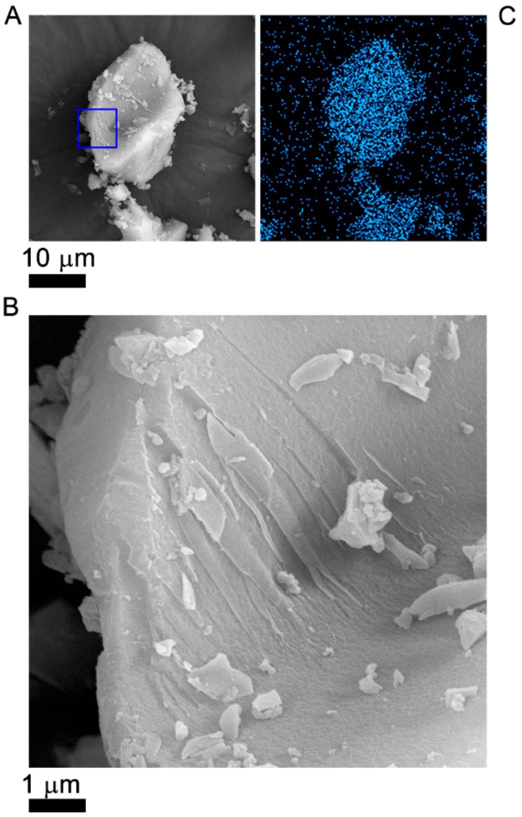

Figure S1. Morphological characterization of the Fe-BTC materials used as matrix in the CNT/Fe-BTC composite materials. (A) FE-SEM image of Fe-BTC matrix and (B) an enlargement of this micro-particles showing sheet-like morphology. The panel (C) represents the compositional map obtained by EDS of image (A), in which the concentration and location of Fe can be observed.

S2. Basic identification of the morphology of SWCNTs and MWCNTs filler material in the CNT/Fe-BTC composite

The microstructure of both SWCNTs and MWCNTs used as filler in the CNT/Fe-BTC composite materials are characterized by using a Field Emission Scanning Electron Microscope, FE-SEM (Hitachi S-4700). **From Figure S2 (A)**, it's possible observed that the SWCNTs appear aggregated, forming bundles. **Figure 2S (B-C)** show the high-resolution scanning electron microscopy (HR-SEM) images of SWCNTs raw material, revealing that the size distribution of SWCNTs is uniform, and the average diameter is about 40-50 nm (Figure 2C) and a length of several microns. As expected, the average diameter of the MWCNTs is greater than that of the SWCNTs (**Figure 2SD-F**), where the average diameter is in the range of about 200 nm (**Figure 2SF**).

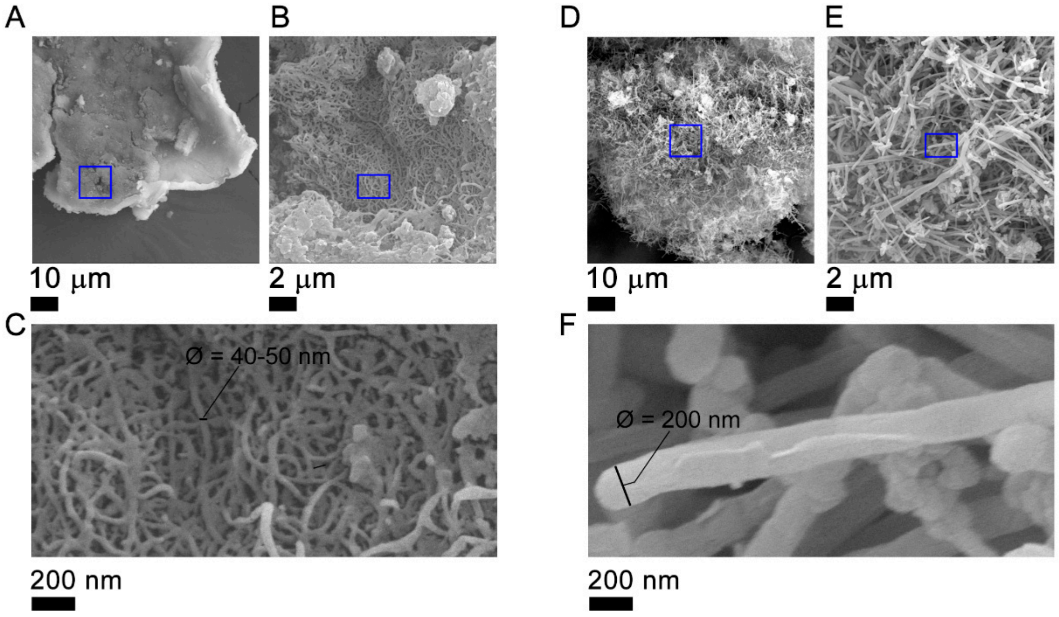

Figure S2. Morphological characterization of the single-walled carbon nanotubes (SWCNTs) and multi-walled carbon nanotubes (MWCNTs) used as filler in the CNT/Fe-BTC composite materials. (A) FE-SEM image of SWCNTs filler and (B-C) an enlargement of these SWCNTs showing their morphology and average diameter. (D) FE-SEM image of MWCNTs filler and (E-F) an enlargement of these SWCNTs showing their morphology and average diameter. The regions indicated by a blue square show the positions where HR-SEM micrographs are taken for each sample.

S3. Determination of Band gap through the Tauc equation.

Figure S3 shows the graph for determinate the band gap in all the materials using the Tauc equation.

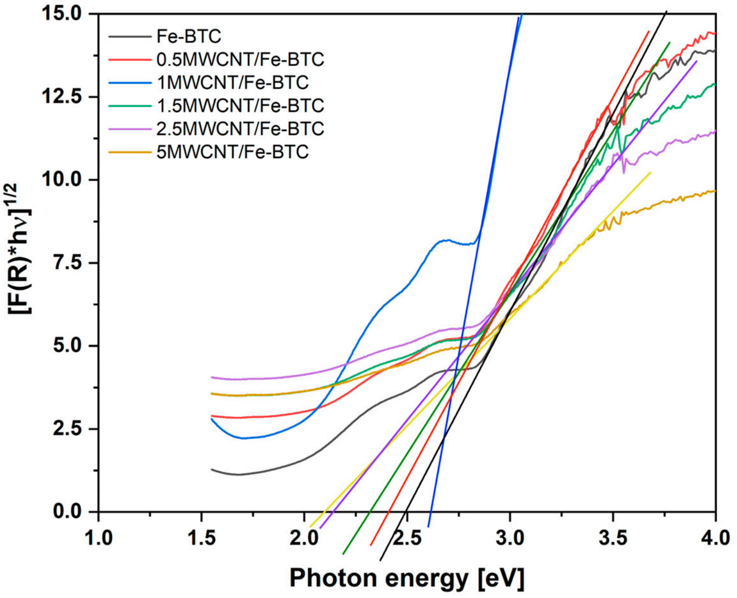

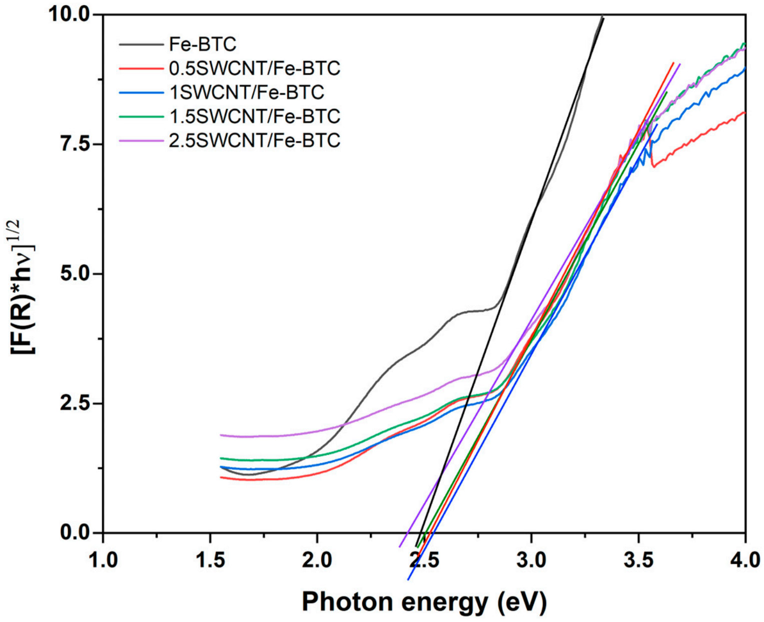

Figure S3. Determination of Band gap through of the Tauc equation. A) MWCNT serie and, B) SWCNT serie.

S4. Raman spectra of the materials used

Figure S4 shows the Raman spectra of the materials used in the Co2-photocatalytic reduction reaction for both series. As can be observed, all the Raman spectra show the bands characteristics of Fe-BTC fresh indicated the stability of MOF Fe-BTC. This same behavior was observed for both series.

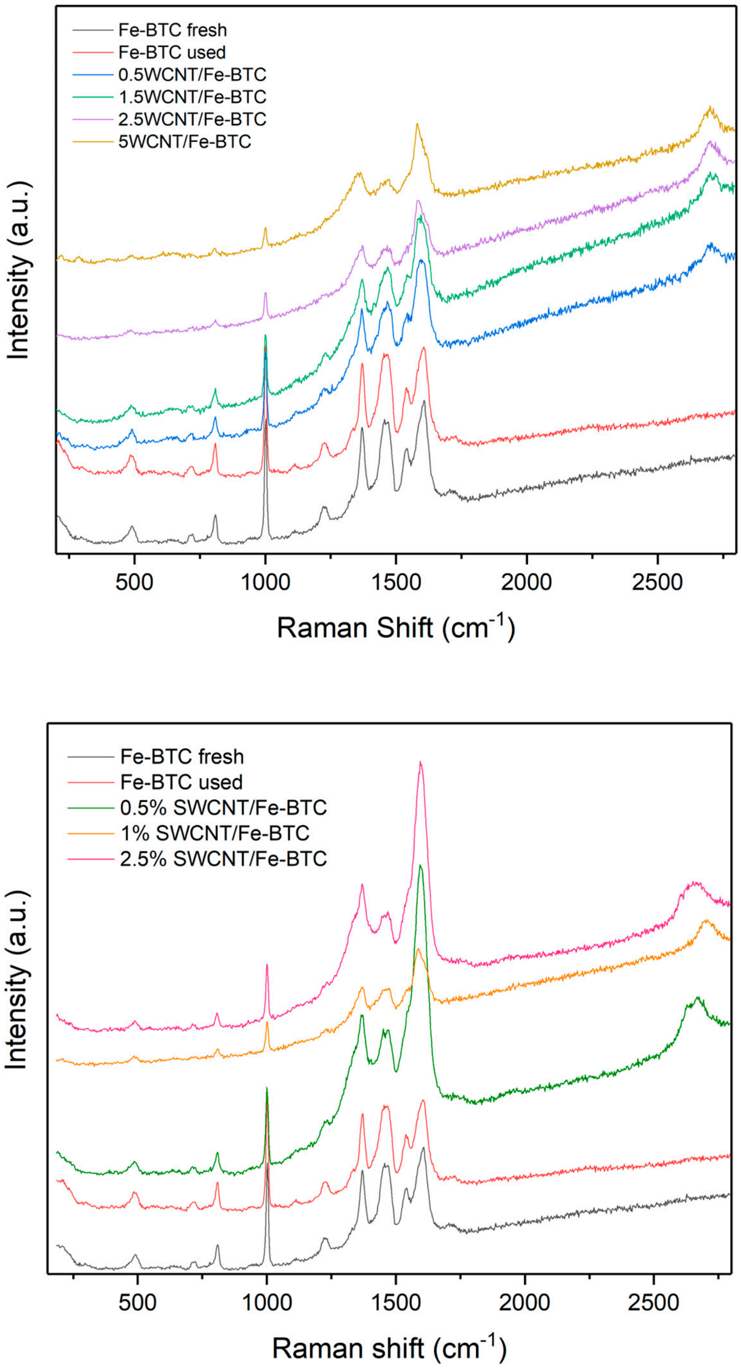

Figure S4. Raman Spectra of the MOF Fe-BTC and composite materials used in the CO2- photocatalytic reduction reaction for both series, A) MWCNT serie, and B) SWCNT serie.
